# Shared versus non-shared prepulse perfusion MR sequence in absolute myocardial perfusion quantification

**DOI:** 10.1186/1532-429X-17-S1-P43

**Published:** 2015-02-03

**Authors:** Kaatje Goetschalckx, Frank E Rademakers, Jan Bogaert, Attila Toth, Béla Merkely, Stefan Janssens, Piet Claus

**Affiliations:** 1 Cardiovascular Diseases, University Hospital Leuven, Leuven, Belgium; 2 Department of Cardiovascular Sciences, KU Leuven, Leuven, Belgium; 3 Department of Radiology, University Hospital Leuven, Leuven, Belgium; 4 Heart and Cardiovascular Center, Semmelweis University, Budapest, Hungary

## Background

First-pass perfusion cardiac magnetic resonance (CMR) allows the quantitative assessment of myocardial blood flow (MBF). Currently, clinical translation is lacking, mainly due to considerable disparity in quantification methodology. The aim of this study was to compare shared (SP) and non-shared (NSP) prepulse perfusion MR sequences for MBF quantification.

## Methods

In this substudy of the NOMI-trial (ClinicalTrials.gov identifier: NCT01398384), quantitative perfusion analysis was compared between patients that underwent an MR first pass perfusion sequence with a shared (n=25) and a non-shared prepulse (n=25), at 4 months after revascularized myocardial infarction. Perfusion imaging consisted of 3 short-axis slices acquired every heartbeat, with a balanced turbo gradient echo sequence in a 1.5 tesla MR unit (Achieva, Philips Medical Systems, The Netherlands) and was performed at rest and during adenosine (140 µg/kg/min) stress. MBF was quantified using Fermi deconvolution with a single bolus (SB, 0.05 mmol/kg) and dual bolus (DB, equal volumes of 0.0027 mmol/kg followed by 0.05 mmol/kg of contrast agent) analysis technique, in 6 segments of basal and midventricular short axis slices. Apical segments and segments with infarct scar on the corresponding late gadolinium enhancement-images were excluded.

## Results

Baseline characteristics of both patient groups were comparable. MBF values were significantly higher with SP than with NSP sequence for the SB analysis technique. For DB, MBF was not different for rest perfusion, but MBF during stress perfusion was significantly lower with SP than with NSP sequence. At a contrast dose of 0.05 mmol/kg, the relationship between signal intensity and contrast agent concentration is subject to saturation in both the left ventricular (LV) luminal and myocardial signal intensity curves. In the SB technique, saturation of the LV luminal signal intensity curve predominates the analysis result, with overestimation of MBF. In the DB technique, saturation of the myocardial signal intensity curve becomes relevant when MBF is high (stress perfusion), resulting in underestimation of MBF.

## Conclusions

To minimize saturation effects in myocardial perfusion quantification, a NSP MR sequence is preferable over SP. Both SB and DB analysis techniques are subject to saturation, especially at high MBF. Future MR sequence developments towards more stable and non-saturated signal intensities are needed.

## Funding

No disclosures.

**Figure 1:**
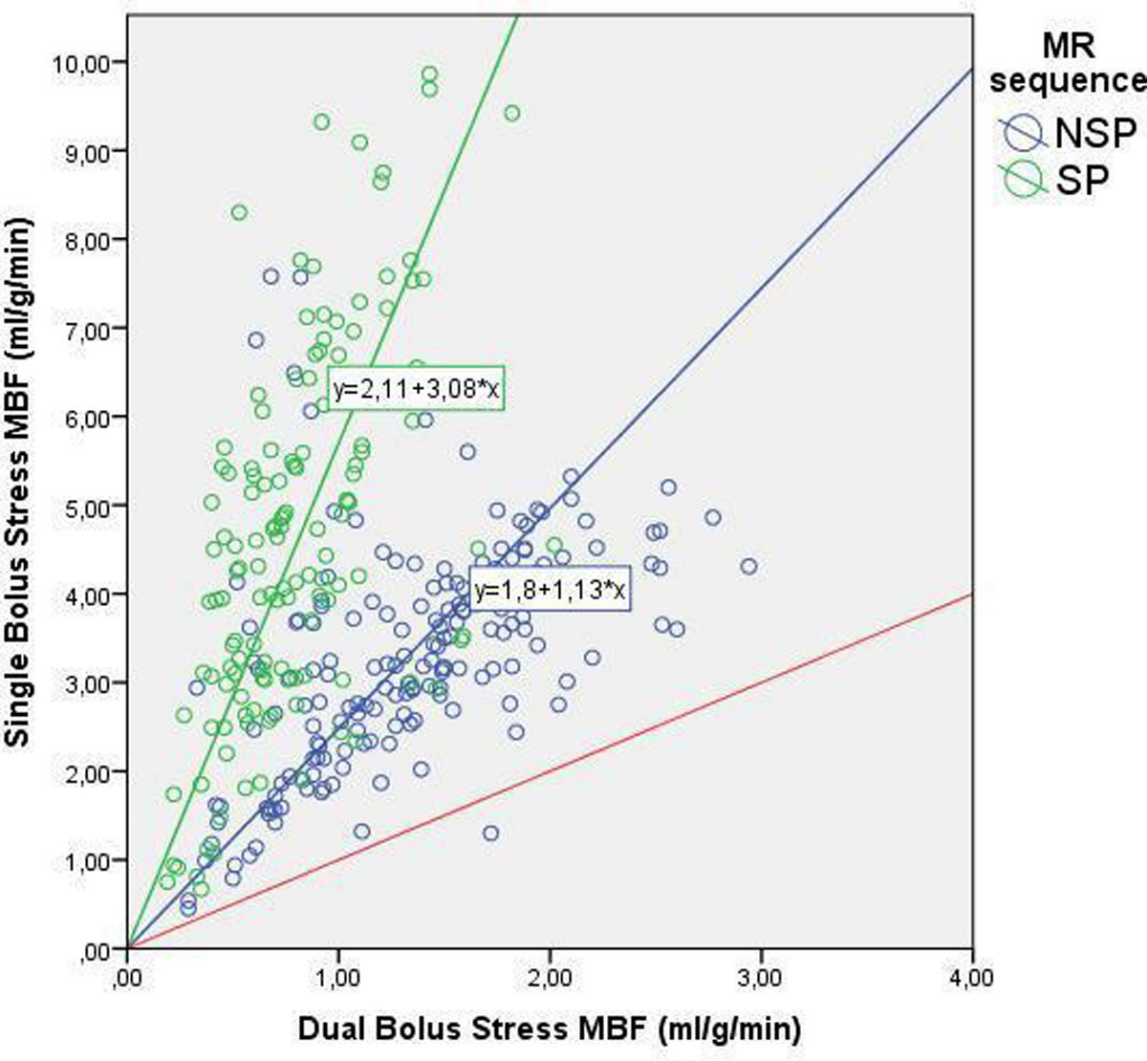
Dots represent absolute MBF in ml/g/min with the dual bolus (X-axis) and the single bolus analysis technique (Y-axis) during adenosine stress perfusion. NSP MR sequence (blue dots) results in higher MBF values with the SB than with the DB analysis technique (blue line shifted to the left of the red equality line), and this is even more the case for the SP MR sequence (green dots and line). In the absence of a saturation effect, theoretically the MBF value calculated with the DB and SB analysis technique would be the same (red equality line).

**Table 1:** 

MBF (ml/g/min)	SP	NSP	p
mean ± SD (n)			

Rest			

SB	2.57 ± 1.36 (160)	1.58 ± 0.60 (168)	< 0.05

DB	0.41 ± 0.21 (192)	0.40 ± 0.26 (175)	ns

Stress			

SB	4.64 ± 2.05 (148)	3.09 ± 1.15 (163)	< 0.05

DB	0.82 ± 0.35 (174)	1.37 ± 0.54 (163)	< 0.05

MPR			

SB	2.17 ± 1.37 (135)	2.27 ± 1.26 (156)	ns

DB	2.41 ± 1.38 (169)	4.61 ± 2.39 (163)	< 0.05

MPR = myocardial perfusion reserve = MBF stress/ MBF rest

